# Berberine inhibits enterovirus 71 replication by downregulating the MEK/ERK signaling pathway and autophagy

**DOI:** 10.1186/s12985-016-0674-4

**Published:** 2017-01-11

**Authors:** Huiqiang Wang, Ke Li, Linlin Ma, Shuo Wu, Jin Hu, Haiyan Yan, Jiandong Jiang, Yuhuan Li

**Affiliations:** 1 Institute of Medicinal Biotechnology, Chinese Academy of Medical Sciences and Peking Union Medical College, Beijing, 100050 China; 2 Institute of Materia Medica, Chinese Academy of Medical Sciences and Peking Union Medical College, Beijing, 100050 China

**Keywords:** Enterovirus 71 (EV71), Berberine, Antiviral activity, Autophagy, MEK/ERK signaling pathway

## Abstract

**Background:**

The MEK-ERK signaling pathway and autophagy play an important role for enterovirus71(EV71) replication. Inhibition of MEK-ERK signaling pathway and autophagy is shown to impair EV71 replication. Berberine (BBR), an isoquinoline alkaloid isolated from *Berberis vulgaris L.*, has been reported to have ability to regulate this signaling pathway and autophagy. Herein, we want to determine whether berberine can inhibit EV71 infection by downregulating the MEK/ERK signaling pathway and autophagy.

**Methods:**

The antiviral effect of berberine was determined by cytopathic effect (CPE) assay, western blotting assay and qRT-PCR assay. The mechanism of BBR anti-virus was determined by western blotting assay and immunofluorescence assay.

**Results:**

We showed that berberine does-dependently reduced EV71 RNA and protein synthesis, which was, at least in part, the result of inhibition of activation of MEK/ERK signaling pathway. Furthermore, we found that berberine suppressed the EV71-induced autophagy by activating AKT protein and inhibiting the phosphorylation of JNK and PI3KIII.

**Conclusions:**

BBR inhibited EV71 replication by downregulating autophagy and MEK/ERK signaling pathway. These findings suggest that BBR may be a potential agent or supplement against EV71 infection.

## Background

Enterovirus, which belongs to the *Picornaviridae* family, is often associated with a serious infectious disease affecting millions of people worldwide. In particular, Enterovirus 71 (EV71) is the most common cause for hand, foot and mouth disease (HFMD) in children under the age of five [1]. EV71 was first isolated from patients in California in 1969. Since then, its outbreaks have been periodically reported worldwide, especially in the Asia-Pacific region. EV71-caused HFMD is often associated with severe neurological diseases and fatalities. Unfortunately, currently there are no effective antiviral drugs in the clinic to treat EV71-induced HFMD [2, 3].

It is known that viruses successfully infect host cells, needing toutilizemany functional components of different cellular signaling pathways in cells. Mitogen activated protein kinases (MAPKs) are important molecules mediating innate immunity in viral infection and activation of the MEK/ERK MAPK signaling pathway has been shown to be essential for EV71 replication in embryonic rhabdomyosarcoma (RD) cells [4, 5], human embryonic kidney (HEK) 293 cells [4], SK-N-SH cells [6] and immature dendritic (iDCs) cells [7]. Inhibition of MEK/ERK signaling pathway by U0126 or specific siRNAs has been found to impair EV71 replication [4–7]. These recent studies strongly supports that MEK/ERK signaling pathway plays an essential role in EV71 life cycle and pathogenesis. Therefore, blockage of MEK/ERK signaling pathway may be an excellent strategy in limiting EV71 infection.

In addition, virus-associated autophagy is also known to provide a support for viral replication. Much evidence shows that viral infection induces autophagy in host cells including human cytomegalovirus [8], hepatitis C virus [9], herpes simplex virus type I [10], coxsackievirus B3 [11], influenza A virus [12], human immunodeficiency virus type I [13], and EV71 [14–16]. Autophagy induced by viruses infection may provide a support for viruses replication. It has been reported that EV71 infection could induce autophagic machinery to promote EV71 replication in vivo and in vitro [14]. Fu et al. demonstrated that EV71 infection African green monkey kindy cells (Vero) induced autophagy and they identified a miRNA, miR-30a, that inhibited EV71 replication by modulating EV71-induced autophagy [16]. Lee et al. found that EV71-induced autophagy increases viral replication and pathogenesis in a suckling mouse model [17]. Therefore, therapeutic modulation of autophagy may be a promising strategy for inhibiting EV71 replication.

**Fig. 1:**
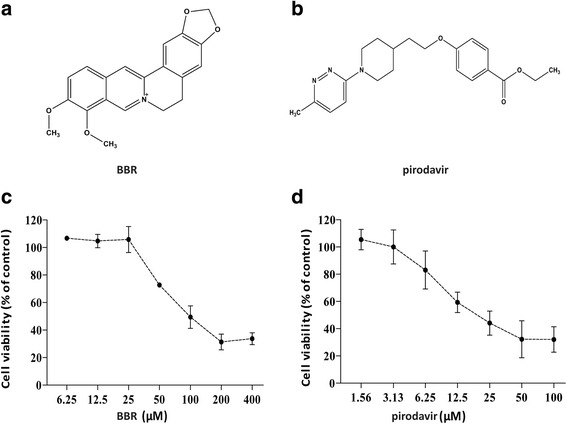
The chemical structure of compounds and CCK assay. **a** The chemical structure of BBR. **b** The chemical structure of pirodavir. **c** The CCK assay of BBR. **d** The CCK assay of pirodavir

Berberine (BBR, Fig. 1a), an isoquinoline alkaloid isolated from *Berberis vulgaris L.*, has been reported to have multi pharmacological effects such as antidiarrheal effect [18], antibacterial effect [19], hypotensive effect [20], and antiviral activity [21, 22]. Most of these pharmacological effects is known to be associated with the mechanisms that BBR regulates signaling pathway in vitro, such as, EGFR/MEK/ERK signaling pathway [23], NF-κB pathway [22], AMPK/mTOR signaling pathway [24]. In addition, Deng et al. demonstrated that BBR attenuates autophagy in adipocytes by targeting BECN1 [25]. These findings suggest that BBR can regulate MEK/ERK signaling pathway and autophagy, which implies that BBR might be effective in limiting viral replication. In line with this notion, BBR is shown to inhibit the replication of respiratory syncytial virus (RSV), herpes simplex virus (HSV), human papilomavirus (HPV), and human cytomegalovirus (HCMV) [21, 22, 26, 27]. However, it is unclear that BBR has an inhibitory action against EV71. Herein, the aim of our research is to determine whether BBR inhibits EV71 replication by downregulating autophagy and MEK/ERK signaling pathway.

## Results

### BBR inhibits EV71 replication

To determine the anti-EV71 activities of the tested compounds, their abilities to inhibit EV71-induced CPE in Vero cells. First, we first detected the toxicity of BBR and pirodavir in Vero cells by Cell Counting Kit (CCK) assay (Fig.1c, d). As shown in Table 1, the 50% toxicity concentration (TC_50_) of BBR was 73.10 μmol/L in Vero cells and the TC_50_ of pirodavir (Fig. 1b) was 27.49 μmol/L in Vero cells.

**Table 1:** The efficiency of BBR and pirodavir against EV71 in vitro

	Pirodavir			BBR		
	TC_50_ (μM)	IC_50_ (μM)	SI	TC_50_ (μM)	IC_50_ (μM)	SI
EV71(H)	27.49 ± 5.18	0.41 ± 0	67.05	73.10 ± 0.79	8.55 ± 0	8.55
EV71(SHZH98)	27.49 ± 5.18	0.26 ± 0.05	105.73	73.10 ± 0.79	10.25 ± 7.24	7.13
EV71(JS-52)	27.49 ± 5.18	0.47 ± 0.08	58.49	73.10 ± 0.79	7.43 ± 1.59	9.84
EV71(BrCr)	27.49 ± 5.18	0.22 ± 0.11	124.95	73.10 ± 0.79	7.43 ± 1.59	9.84

Values provided in this table represent the mean of three independent experiments

Next,we performed CPE assays according to the above results. We found that BBR significantly inhibited the replication of all the tested strains, including H, JS-52, SHZH98 and BrCr stains, with the 50% inhibitory concentration (IC_50_) values ranging from 7.43 to 10.25 μM (Table 1).

**Fig. 2:**
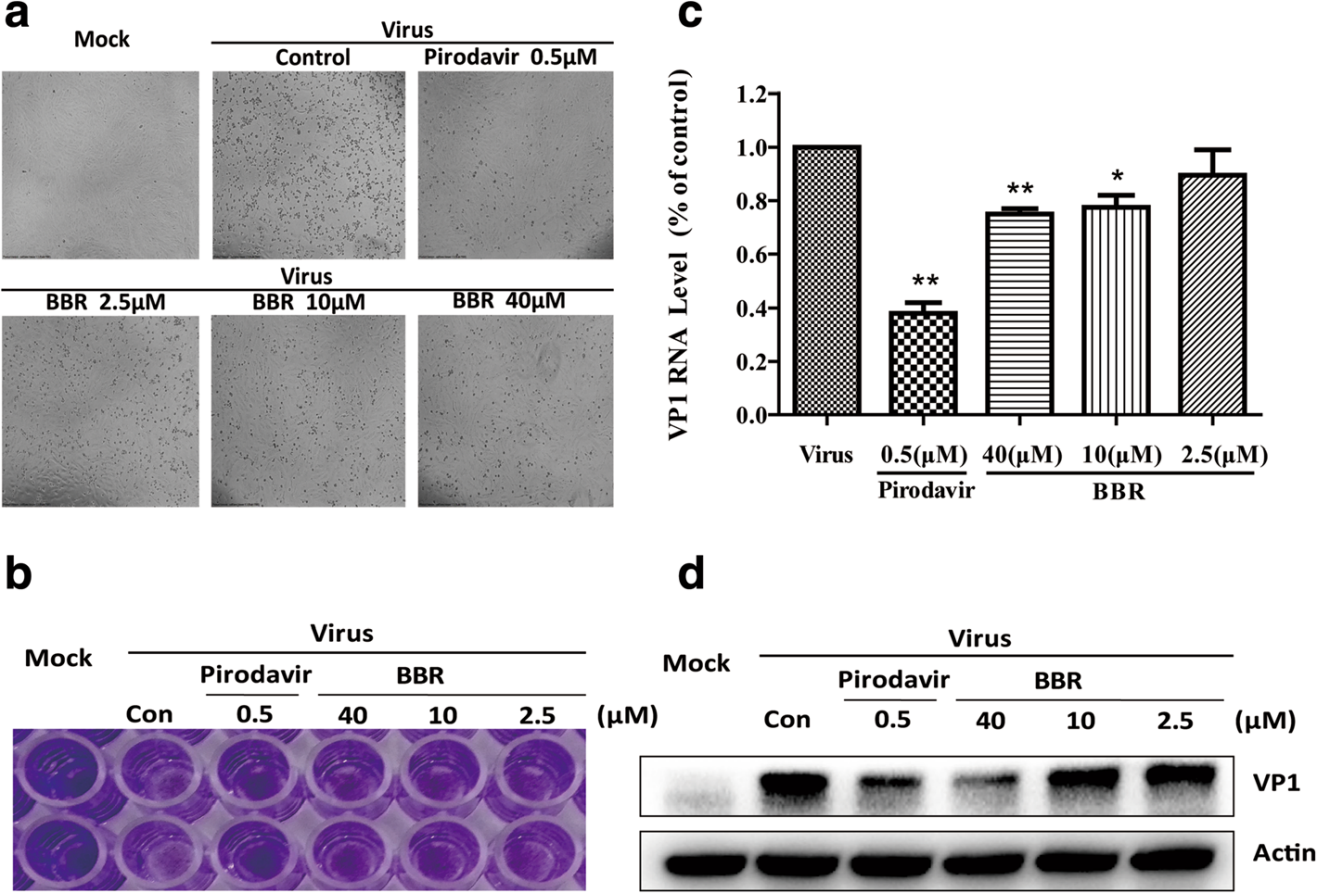
The antiviral effect of BBR and pirodavir against EV71 in Vero**. a** BBR and pirodavir reduced the EV71-induced CPE in Vero cells. Cells were examined using a microscopy (×40). **b** BBR and pirodavir reduced the EV71-induced CPE in Vero cells. Cells were examined using crystal violet staining. **c** BBR and pirodavir reduced the expression of EV71 VP1 RNA by one-step qRT-PCR assay. **P < 0.001 *P < 0.05 **d** BBR and pirodavir reduced the expression of EV71 VP1 protein in Vero cells by western blot assay

Similarly, the activity of BBR anti-EV71 virus also visually demonstrated with electron microscope photograph and crystal violet staining in Vero cells (Fig.2a and b). To futher confirm the inhibitory action of BBR and pirodavir against EV71, the expression of VP1 and EV71 capsid protein, was analyzed to examine its effect on EV71 biological synthesis. As shown in Fig. 2d, BBR decreased the expression of VP1 protein in a dose dependent manner. In the same way, BBR treatment dose-dependently decreased the level of VP1 RNA measured by reverse transcription-quantitative polymerase chain reaction (RT-qPCR) assay (Fig. 2c). The reference drug Pirodavir also significantly decreased the RNA and protein synthesis of EV71. Collectively, BBR demonstrated a potent inhibitory activity against EV71 virus.

### BBR inhibits the phosphorylation of MEK/ERK signaling pathway

**Fig. 3:**
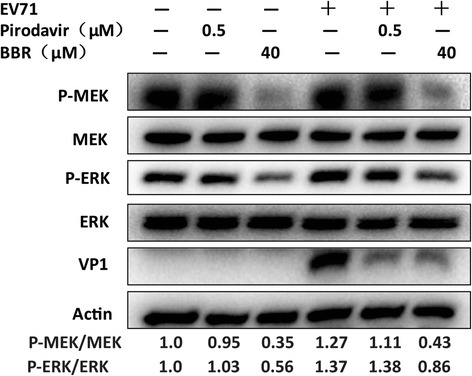
BBR but not pirodavir reduces the phosphorylation of MEK/ERK. Vero cells (9 × 10^5^ cells/well) were plated into 6-well culture plates. Vero cells were mock-infected or infected with EV71 (H, MOI = 0.1) for 1 h. The cells were then treated with BBR (40 μmol/L) and pirodavir (0.5 μmol/L), respectively, for 24 h. The cells were harvested and proteins were examined by western blot

It was known that MEK/ERK were downstream components of epidermal growth factor receptor (EGFR) signaling pathways which can be activated by several stimulus including EV71 infection in various cell types [6, 28]. Inhibition of MEK/ERK signaling pathway by U0126 or specific siRNAs has been found to impair EV71 replication [4–7] and BBR was reported to regulate this signaling pathway in vitro. Thus, we assessed whether BBR affected EV71 replication by regulating the phosphorylation of MEK/ERK. As shown in Fig.3, both BBR and poridavir inhibited EV71 replication. However, the phosphorylation of MEK/ERK was significantly attenuated by treatment with BBR but not pirodavir.

### BBR inhibits EV71-induced autophagy

Autophagy induced by virus infection provides a support for viral replication. It has been reported that EV71 infection could induce autophagic machinery to promote EV71 replication in vivo and in vitro [14]. JNK signaling pathway plays an important role in regulation of cell growth, proliferation, differentiation, migration and apoptosis. Studies have shown that JNK signaling pathway is closely related to autophagy, and inhibition of JNK signaling pathway could inhibit autophagy. PI3KIII, as an upstream activator of autophagy, plays an important role in autophagy.

**Fig. 4:**
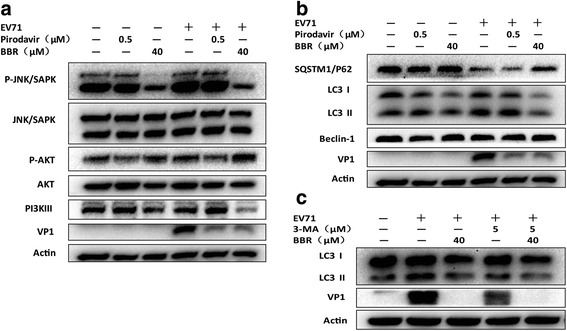
BBR could inhibit EV71-induced autophagy. (**a**, **b**) BBR but not pirodavir can reduce EV71-induced autophagy. Vero cells were mock-infected or infected with EV71 (H, MOI = 0.1) for 1 h. The cells were then treated with BBR (40 μmol/L) and pirodavir (0.5 μmol/L), respectively, for 24 h. The cells were harvested and proteins were examined by western blot. (**c**) BBR and 3-MA could both reduce EV71 infection by inhibiting autophagy. Vero cells were mock-infected or infected with EV71 (H, MOI = 0.1) for 1 h. The cells were then treated with BBR (40 μmol/L) and 3-MA (5 μmol/L), respectively, for 24 h. The cells were harvested and proteins were examined by western blot

**Fig. 5:**
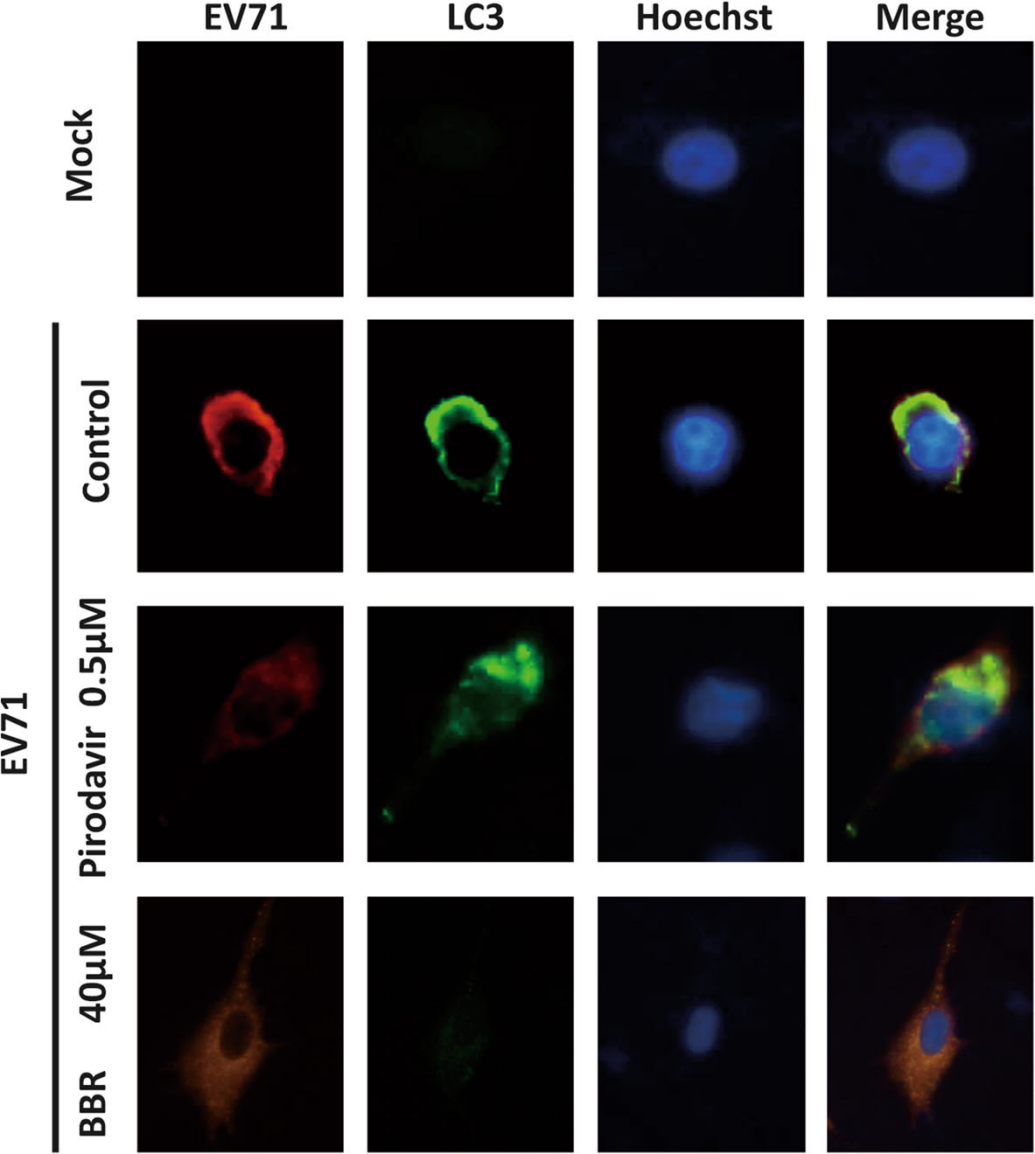
BBR could inhibit EV71-induced autophagy. BBR but not pirodavir can reduce EV71-induced autophagy by immunofluorescence assay. Vero cells were mock-infected or infected with EV71 (H, MOI = 0.1) for 1 h. The cells were then treated with BBR (40 μmol/L) and pirodavir (0.5 μmol/L), respectively, for 24 h. Cells were examined using a fluorescence microscopy (×400)

Otherwise, AKT, as an upstream regulator of autophagy, is usually considered to be an autophagy inhibitor. We speculated that BBR inhibited EV71-induced autophagy by affecting JNK, PI3KIII and AKT signaling pathway. As shown in Fig.4a, BBR increased AKT phosphorylation and reduce JNK and PI3KIII phosphorylation. Accordingly, we found that the lapidated LC3BII, a marker for autophagy, decreased in the presence of BBR. Consistent with this, the amount of SQSTM1/P62 increased in BBR-treated cells compared to virus control, which suggested that BBR was effective in inhibiting autophagy. However, BECN-1 expression was not affected by BBR (Fig.4b). To further confirm the result of BBR inhibition of autophagy, we next used fluorescence microscopy to observe the effect of BBR on EV71-induce autophagy. As shown in Fig.5, LC3B expression was inhibited by BBR not pirodavir in EV71-infected Vero cells. Similarly, 3-MA, an autophagy suppressor, could also inhibit EV71 replication (Fig.4c). Therefore, EV71-induced autophagy in Vero cells was attenuated by BBR but not pirodavir although both two compounds could inhibit EV71 replication.

## Discussion

EV71, a member of the *Picornaviridae* family, causes HFMD by spreading through contact with virus-containing body fluids, respiratory droplets, and feces. However, there are no effective antiviral drugs available for the treatment of HFMD. Some natural medicinal compounds have been demonstrated to be active against the disease by ameliorating the symptoms and shortening the course [29, 30]. Three capsid-targeting molecules known as “WIN” compounds are currently in clinical development [31, 32]. According to our results (date not shown), the antiviral activity of pirodavir against EV71 is better than pleconaril. Thus, we used pirodavir as positive control for the evaluation of the antiviral activity of BBR.

BBR has been reported to have multi pharmacological effects such as antibacterial effect [19], hypotensive effect [33], and antiviral activity [21, 22]. It has been reported that BBR could regulate signaling pathway in vitro, such as, EGFR/MEK/ERK signaling pathway [23], AMPK/mTOR signaling pathway [24]. It is interesting thing that BBR should promote EV71 replication since BBR activated ERK, which is required for the viral replication. However, BBR exhibited inhibited activity against EV71 in a dose-dependent manner. BBR inhibited the activation MEK/ERK signaling pathway in Vero cells. Also, Liu et al. found that BBR induced senescence of human glioblastoma cells by downregulating the EGFR/MEK/ERK signaling pathway [23]. We thus think that BBR may have different effects on the same signaling pathway in different cell lines.

Otherwise, the preliminary analysis indicated that BBR inhibited EV71-induced autophagy. BBR suppressed LC3BII in both normal Vero cells and EV71-infected Vero cells. These findings suggest that BBR inhibits EV71 replication,at least partly by inhibiting autophagy. Of note, several studies showed that BBR also upregulated autophagy in some cell lines such as J774A.1 cells [24] and HepG2 cells [34]. Obviously, it is very possible that BBR has different effect on autophagy in different cell lines.

Overall, our research is the first time to report the anti-EV71 mechanism of BBR and our findings provide a new clue for developing the anti-EV71 drug by inhibiting MEK/ERK signaling and EV71-induced autophagy. However, many questions remain to be solved, e.g., whether berberine is effective in limiting EV71 infection in vivo. In future, we would further explore these questions in mouse models prior to clinical trials in further studies.

## Conclusions

In this study, we found that BBR inhibited EV71-induced autophagy and the activation of MEK/ERK signaling pathway. These findings suggest that berberine might be a potential lead or supplement for the development of new anti-EV71 agent in the future.

## Methods

### Cells and viruses

African green monkey kidney (Vero) cells were purchased from the American Type Culture Collection (ATCC), and were cultured in Minimum Essential Medium (MEM) supplemented with 10% fetal bovine serum (FBS) (GIBCO) and antibiotics (100 U/ml penicillin and 100 mg/ml streptomycin) at 37 °C in a 5% CO_2_ incubator.

EV71 strain SHZH98 isolated from the throat swab sample of an HFMD case occurring in 1998 in China was kindly provided by Dr. Qi Jin, Institute of Pathogen Biology, Chinese Academy of Medical Science and Peking Union Medical School, Beijing, China. EV71 strain BrCr (VR-1775) and H (VR-1432) were purchased from the ATCC. EV71 strain JS-52 was a kind gift from Dr. Xiangzhong Ye, Beijing Wantai Biological Pharmacy Enterprise Co., Ltd. EV71 were all passaged in Vero cells.

### Compounds

BBR was purchased from the Sigma-Aldrich (St Louis, MO, USA) and the purity is no less than 99%. Pirodavir was purchased from Biochempartner (Shanghai, China). Both BBR and piradavir were dissolved into DMSO.

### Cytotoxicity assay

Cytotoxic effects of BBR on Vero cells were assayed by CCK (TransGen Biotech, China) assay. Briefly, cells (3 × 10^4^ cells/well) were seeded into 96-well culture plates and were incubated overnight. Then, the medium was removed and different concentrations of BBR were applied in triplicate. After 3 days’ incubation, the cytotoxicity of BBR was determined by CCK assay. The signals were read at 450 nm on Enspire (Perkin Elmer,Waltham, MA, USA). The TC_50_ was defined as the concentration that inhibits 50% cellular growth in comparison with the controls.

### CPE inhibition assay for anti-EV71

The anti-EV71 activity of BBR was assayed by CPE inhibition method. Briefly, cells (3 × 10^4^ cells/well) were plated into 96-well culture plates for incubation of 24 h. The medium was then removed and cells were infected with EV71 of 100TCID_50_ (50% tissue culture infective doses) in serum-free medium for 1 h at 37 °C. Then, the unbound viruses were removed and various concentrations of BBR were supplemented for incubation of another 48 h. The IC_50_ defined as the minimal concentration of inhibitor required to inhibit 50% of CPE was determined by Reed & Muench method. The selectivity index (SI) was calculated as the ratio of TC_50_/IC_50_ . In addition, the cells were stained with 0.5% crystal violet in 20% ethanol for 15 min at room temperature and the cells were imaged after rinsed with PBS.

### Western blot analysis

The cells were lysed in the M-PER mammalian protein extraction reagent (Thermo, Rockford, IL) containing halt protease inhibitor single-use cocktail (Thermo). The protein concentration was determined by the BCA reagents (Thermo). About 15 μg proteins were denatured and applied to sodium dodecyl sulfate-polyacrylamide gel electrophoresis (SDS-PAGE). The electrophoresis products were transferred to a polyvinyl idenefluoride (PVDF) film and PVDF membranes were then incubated at room temperature with specific primary antibody. After a standard washing, membranes were incubated with horse radish peroxidase (HRP)-labeled secondary antibody. The assay developed using a chemiluminescent substrate. The primary antibodies used in this study included antibodies against β-actin, p-p44/p42 MAPK, p44/p42 MAPK, p-MEK, MEK, p-AKT, AKT, p-JNK, JNK, PI3KIII, SQSTM1/P62, LC3B, Beclin-1 (Cell Signaling Technology), and EV71-VP1 (Abnova). The goat anti-rabbit and anti-mouse HRP-labeled antibodies were obtained from Cell Signaling Technology.

### Immunofluorescence assay

Vero cells grown on glass coverslips (Thermo) were infected with EV71 (H, MOI = 0.1) for 1 h. Then, BBR and pirodavir were supplemented for incubation of another 24 h. After incubation, the culture medium was removed and the cells were washed and fixed. The cells were permeabilized in 0.5% Triton X-100 at room temperature for 15 min and blocked in PBS containing 1% BSA for 60 min at room temperature. Cells were then incubated with an anti-EV71 antibody (Millipore) and LC3B (Cell Signaling Technology) antibody at a dilution of 1: 500 for 2 h at room temperature. After washing three times with PBS, the samples were reacted with PE conjugate goat anti-mouse secondary antibody (TransGen Biotech, China) and FITC conjugate goat anti-rabbit secondary antibody (TransGen Biotech, China) at a dilution of 1: 500 for 1 h at room temperature. After washing with PBS, add Hoechst (Beyotime Institute of Biotechnology, China) for 10 min and images were taken using a fluorescence microscope (Olympus, IX71).

### Quantitative reverse-transcription polymerase chain reaction (qRT-PCR) quantification

Vero cells (9 × 10^5^cells/well) were plated into 6-well culture plates for incubation of 16 h. The medium was removed and cells were infected with EV71 (H, MOI = 0.1). After 1 h, various concentrations of BBR were supplemented for incubation of another 24 h. The total RNA of the infected cells was extracted using the RNeasy Mini kit (QIAGEN) according to the manufacturer’s instructions. The one-step qRT-PCR was performed with SuperScript III Platinum SYBR Green One-step RT PCR Kit (Invitrogen) using the ABI 7500 Fast Real-Time PCR system (Applied Biosystems). The mRNA expression of EV71 VP1 was detected with sense primer 5′- GATATCCCACATTCGGTGA -3′ and antisense primer 5′- TAGGACACGCTCCATACTCAAG -3′ targeting a conserved region of the VP1 gene. The β-actin mRNA was detected using sense primers 5′-TGACGGGGT CACCCACA CTGTGCCCATCTA-3′ and antisense primer 5′-CTAGAAGCATTTG CGGTGGACG ATG-3′. PCR assay was carried out in a 25 μL volume and the target fragment amplification was carried out as follows: reverse transcription at 50 °C for 3 min; initial activation of HotStar Taq DNA Polymerase at 95 °C for 10 min; 40 cycles in two steps: 95 °C for 15 s, 60 °C for 30s. The relative amounts of EV71 VP1 mRNA was calculated by comparative Ct method after normalizing the quantity of β-actin.

### Statistical analysis

Data are expressed as the mean ± standard error of the mean and analyzed using two-tailed Student’s *t*-tests with *P < 0.001* and *P < 0.05* taken as significant.

## Abbreviations

BBR: Berberine
CNS: Central nervous system
CPE: Cytopathic effect
EV71: Enterovirus 71
HFMD: Hand foot and mouth disease
IC_50_: 50% inhibitory concentration
SI: Selectivity index
TC_50_: 50% toxicity concentration
